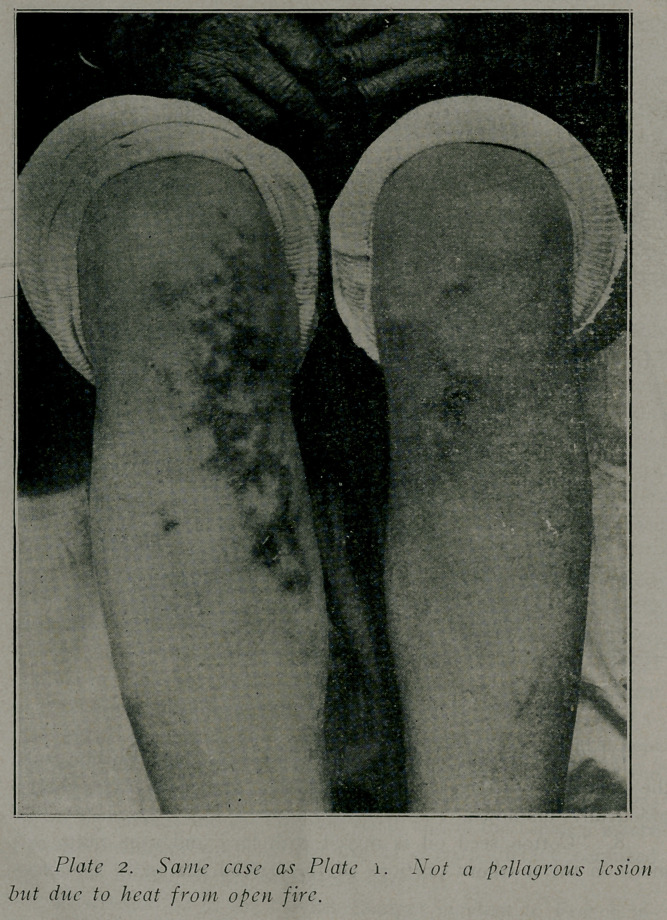# Etiology, Pathology and Treatment of Pellagra

**Published:** 1912-05

**Authors:** Geo. C. Mizell

**Affiliations:** Atlanta, Ga.; Gastro-Enterologist to Wesley Hospital: Formerly Associate Professor of Physiology and Gastro-Enterology of Atlanta College of Physicians and Surgeons


### ETIOLOGY, PATHOLOGY AND TREATMENT OF PELGRA.

By Geo. C. Mizell, M. D.

*Gastro-Enterologist to Wesley Hospital; Formerly Associate Professor of Physiology and Gastro-Enterology of Atlanta College of Physicians and Surgeons.*

One of the propositions set forth as a basis for the theory that cottonseed oil is the cause of Pellagra in America was that the experiments performed with spoiled corn tended to show that the results obtained were due to the oxidized (fermented) oil of corn. As bearing on this proposition is submitted a paper read before the Medical Association of Georgia, April 17, 1912.

### Experimental Pellagra.

In all experimental work to show the cause of pellagra, extracts of spoiled corn or the organisms from spoiled corn have been used. Much labor has been expended to identify the specific agent. Various results have been obtained and,—as Sir Patrick Manson comments, "One fact stands out very prominently and that is that each investigator claims to have reproduced true pellagra, either in animals or men, sometimes in himself, by inoculating beneath the skin, injecting into the veins or administering per orem the special organisms or toxic products which he happens to have isolated."

Nevertheless, the experimental work done by scientific men has been sufficient to show to their satisfaction and to convince many others that pellagra has been produced experimentally.

Out of the confusion of evidence arises the question, which, if either, of the suspected agents—the chemicals or organisms—is the true cause of the disease? Passing over this question by stating that it appears that the weight of evidence is in favor of a chemical agent and that this view is the one most generally accepted by the disciples of the maize theory, we ask, what then is the nature of the chemical? Briefly stated, the method of obtaining the toxic substances of spoiled corn is as follows: The corn is placed in a vessel one-third filled with water and carried slowly through the acetic, alcoholic, lactic and putrid states of fermentation. It is then dried, ground and extracted with 40 per cent. alcohol. The tincture thus obtained is evaporated on a water bath. The residue contains the toxic substances. In this process no certain organism is mentioned nor any particular organism seems necessary. These toxic substances have been designated as: 1st, The Red Oil of Spoiled Corn; 2nd, The Toxic Substance of Spoiled Corn, or Pellagrosine, and, 3rd, The Resinous Substance of Spoiled Corn.

The first and third of these substances are clearly of an oleogenous nature and the second, while no intimation of its nature is given, two of the tests to which it was submitted seem to exclude the possibility of an alkaloid. These tests are, it is soluble in a solution of caustic potash, and from this solution it is precipitated by sulphuric acid; another significant reaction is that it produces a green color with copper sulphate.

It is claimed that a poisonous alkaloid has been separated from the oil and resinous susbtances, but after the alkaloid has been separated the oil and resinous substance is still poisonous in small doses—"a thing which proves that the alkaloid is not the only poisonous substance in spoiled corn." The source of these poisonous substances, Peschal states, is through the action of the Bacterium Madis on the albuminous and oleogenous constituents of corn. That the character of the fat acted upon is important is shown by the fact that no poisonous substance is developed by the growth of Bacterium Madis upon blood serum or other media. Unfortunately these fatty substances have been little studied, in fact, exact chemical methods for the study of these substances are wanting, as evidenced by the statement of Lewkowistch that the only method of identifying rancid fat is the sense of smell.

In the process of preparation outlined above the oil of corn would, under the influence of air, moisture and the ferments, present in the corn, undergo oxidation or fermentation, giving rise to the oxidation products of oil of corn. These oxidation products are numerous and from the properties given of the extracts, red oil of spoiled corn, pellagrosine and resinous substances may all be derived from the oil of corn through oxidation. Association with albuminous matter may or may not be necessary.

It is no digression from the subject to discuss at this point this conception of the disease, for in doing so the stumbling block to the general acceptance of any of the theories so far advanced may be removed.

Lombroso's theory, which experimentation seems to have proven as conclusively as any proposition in medicine, may be stated as follows: Spoiled corn contains a Toxico-Chemical agent, which if consumed in sufficient amount for a sufficient time will produce pellagra.

The experimental work and this theory classifies the disease as a Toxico-Chemical disease and as such it should resemble toxico-chemical diseases in seasonal incidence in that diseases of this class occur at any period of the year. For example, Berriberri, ergotism, alcoholism have no seasonal incidence.

Furthermore, in poisons of this nature, the symptoms abate or persist, according to the presence or absence of the poison in the food of the patient. It is said that spoiled corn produces blind staggers in horses, which disappaers when the noxious food is withdrawn. Once established the symptoms of pellagra continue and recur irrespective of the spoiled corn in the diet. It is held, and rightly so, that it is the continuous consumption of spoiled corn that produces pellagra. This is shown by all of the experiments performed and it is stated that the Italian pellagrines live exclusively on spoiled corn. Antoniu is said to have produced pellagra in seven people by feeding them exclusively on spoiled corn.

Notwithstanding the mass of authentic evidence, the fact remains that well-sustained grounds have caused many able investigators to reject all theories based upon it. Aside from the peculiar seasonal incidence which is opposed to an exclusive toxico-chemical idea, one fact has not been harmonized with any of the maize theories.

Every writer of note has credited the reports of cases in which corn products could be excluded as an etiological factor. I have had under observation for some months four cases in the same family, only one of whom ever ate corn products in any form. Before further progress can be made in this discussion it is necessary to set forth that there are two distinct types of pellagra, as regards seasonal incidence, namely: one having a seasonal incidence corresponding to hot seasons, and another type occurring independent of climatic influences. Cases of the last type have not been numerous; yet, not a few have come under my observation. During the past winter no less than four cases with well defined pellagrous symptoms have been observed. Two of these cases have been seen by my colleague, Dr. Gaines. That by far the largest number of cases have a summer incidence is not to be denied, and the development of typical pellagra in a season such as the past winter is not without significance. Can it not be held that this behavior of pellagra shows that the cause of each type, while related, must differ materially in some respect? The winter cases show that this disease, in some instances, is uninfluenced by season and in this type the sunlight is not the exciting cause of the dermatitis.

The summer cases show that the disease in many cases is influenced by season and in this type that the sunlight is the exciting cause of the dermatitis.

Go back now to experimental pellagra, which is claimed to be a toxico-chemical disease. It is inconceivable that a poison, found outside of and introduced into the body as such, can limit its effects, becoming active only under the influence of the sun in some cases, yet exhibiting the same effects uninfluenced by the sun in others. Going a step further, may it not be concluded that non-seasonal pellagra alone conforms to the behavior of all other toxico-chemical diseases, in that they do not exhibit a seasonal incidence?

So much for the peculiar seasonal behavior of the incidence as opposed to the exclusive experimental type and passing to those authentic cases who do not eat spoiled corn products, or any other corn products.

Recently there has been an effort made to bridge the chasm and supply the missing link between polenta and pellagra. Without regard to the experimental evidence upon which the doctrine of his faith is founded, the author of the first American book on pellagra accepts the suggestion that no one in this day can truthfully say that he has never eaten corn products. As proof of this statement he indicts corn starch, corn whiskey, corn syrup, corn candy and corn breakfast foods. That such a suggestion is without warrant, your attention is directed to the process outlined above for obtaining the toxic products. This process excludes the possibility of the suspected agents being present in whiskey, even though they are present in mash. If syrup is made from corn carried to the state of putrefaction, it yet remains to be shown that the poisonous substances are soluble in a mixture of glycerine and water.

Dr. C. C. Bass, an able investigator, has had to indict starch in order to connect corn with one of his cases. He states that the patient had for some time eaten a pound of starch a day. It appears reasonable that she must have had pellagra insanity to have contracted such a habit.

Thus, it is easier to admit pellagra without corn than to establish the connection. Nor is it necessary to reject the experimental evidence that agents derived from fermentation or oxidation of oil and albuminous matter of corn does cause pellagra, because some cases of pellagra obtain the same specific poison from another source. Corn has no monopoly on oil or albuminous material, nor is the chemical nature of the oil or albuminous material peculiar to corn. Why, then, should not oils and albuminous materials chemically the same and fermented under the same conditions give rise to the same chemical compounds?

In experimental pellagra the toxic agents are shown to be different from a physical standpoint, but this may be due to different degrees of transformation and not to any marked chemical difference, hence they may arise from the same substances and the essential substance seems to be a certain kind of oil. Following up this suggestion, it will be seen that this oil is present in corn to a much greater degree than in any other edible grain or seed, also that the one suspicious constituent of the oil is Linolin. After identifying linolin as the peculiar constituent of corn that is necessary for the formation of the pellagra-producing agents, it is easy to conclude that its presence in like proportions in other oils will render them capable of forming pellagra-producing agents under the same conditions. Thus may be identified sesame seed, poppy seed, cotton seed, sunflower seed, and many other seed, also some nuts which furnish food stuffs.

Such a conception removes the objection offered by those cases who have not eaten corn and paves the way for a general acceptance of the experimental evidence. The experimental evidence, however, comprehends only one type of pellagra, and this conception of the disease alone is far from satisfactory.

As it is impossible that a chemical poison formed outside of the body can exhibit the peculiar seasonal activity exhibited in the large majority of cases, there remains to be identified a related agent which is capable of exhibiting this seasonal activity.

By studying the experimental evidence and identifying the essential constituent of corn which enters into the formation of the toxico-chemical agent, we may be able to reach a reasonable conclusion as to the cause of the other type of the disease. As before stated, it seems conclusive that this constituent is the neutral fat, linolin.

A brief reference here to the chemical technology of fats and oils shows that linolin has a very wide distribution in nature, and may occur to some extent in animal fat when present in the food of the animal. In contrast with the ordinary animal fats, olein, palmitin and stearin, which are among the most stable constituents in the animal body, linolin is very unstable and readily undergoes fermentation or oxidation. Suppose, then, that the stable oleyl, palmityl and stearyl compounds of the animal body are replaced by the unstable linolyl compounds. Does it not follow that such animals tissue becomes unstable and that there may be developed in the body a bichemical poison related to the "toxico-chemical" poison of experimental pellagra?

In conclusion, I wish to state that while I do not deny the possibility of a type of the disease corresponding to experimental pellagra, not one case has come under my observation that a satisfactory investigation did not show cotton seed oil consumption. For this reason, I am disposed to believe that the consumption of this class of oils, beyond a certain limit, is the essential primary factor, and that seasonal influence is the exciting cause in those cases exhibiting a seasonal incidence, while spoiled corn, or oil expressed from fermented seed, is the exciting cause in those cases uninfluenced by season.

Had the etiology of Pellagra been easy of solution, the question would have been settled long ago. When this theory was proposed, the author expected objections to be offered. Some of the objections proposed deserve some attention, although they have been, in the main, shown to be without facts to support them. In the January to March, 1912, Bulletin of the Georgia State Board of Health, the usual objections with some new ones have been proposed as being sufficient to show that the theory is untenable. Before discussing these objections, the writer wishes to commend the Secretary in his stand for much needed legislation to regulate the importation of corn into the State. This matter is of vital importance to our people. Whether rotten corn is the cause of Pellagra or not, it is certainly unwholesome for both man and stock and as he says, "We are entitled to good corn when we pay our money for it." So the people should follow his suggestion and insist that our law-makers enact adequate measures.

The objections which the Secretary urges will be given in full and such evidence as is in hand will be given to show that they may not be based upon facts or are not opposed to the oil theory. He write:s

1. "It is conceded by every one who has gone into the matter thoroughly that Pellagra did not exist in Spain and Italy prior to the eighteenth century, while the consumption of oils was in all probability quite as great before that time as since."

2. "Oils are too dear, particularly olive oil, to be an article of common diet among the extremely poor peasants of northern Italy and northern Spain, where in the past the disease has flourished."

3. In southern Italy and south Spain, where much more oil is consumed and where the olive tree grows, Pellagra is unknown."

These objections are so closely related that they will be considered together. In regard to the history of Pellagra and oil consumption in Spain and Italy, it may be stated that the exact date is wanting and that general statements are worthless and are of no value when confronted by positive evidence of recent history.

The consumption of seed and nut oils is a part of history which no one can study in connection with the history of pellagra without being impressed with the fact that both are parallel. Oil producing seed flourish in the Mediterranean region and constitute the fat of the poor. Although they may be too poor to buy oil, the material has always been at hand with which to produce it. Since 1817, when the first seed crushing mill was established in Marseilles, there has been a rapid increase in the consumption of this class of oils. Recent information from northern Italy supports the opinion that even the poorer classes eat oils of the same chemical nature of cotton seed oil and that these oils have always been available. The same may be said of Spain. Mr. Brodie, in the Daily Trades and Consular Report, mentions the daily complaint that in northern Spain olive oil is being mixed with other oleogenous substances. While he does not mention the nature of these substances, this statement is enough to show that there are cheap oils in northern Spain.

There is no warrant for the statement that the consumption of oils has remained the same in northern Spain and northern Italy. The consumption of seed oils has been on the gradual increase for a period covering several centuries. This increase has been very rapid in the last century. A common statement emanating from the disciples of the maize theory is that the peasants of northern Italy and northern Spain subsist almost exclusively on corn products. The following information is copied from Pellagra, Marie, Lavinder and Babcock's Translation:

"In Villempenta, a region much affected by Pellagra, the ordinary food consists of wheat cakes cooked with oil or lard, rice, fish and pork, besides corn in the form of pancakes and polenta." "At San Martino, all Argine, a place where Pellagra is frequent, the nourishment is polenta, cheese, sardines, besides annually nine sacks of corn and three of wheat for every able-bodied laborer. At Mel Belluno, fifty per cent. of the whole population is Pellagrous, the food is polenta, cheese, milk, often beans and chestnuts and on feast days, macaroni and rice.

"In the province of Lucca, mostly affected, they eat beef and pork. Ceru gives these statistics:

| | Population | Beef Butchered | Pork Butchered | Total Meat Butchered |
|---|---|---|---|---|
| Porcari | 4621 | 5600 kg | 26333 kg | 31,933 |
| Capenneri | 4222 | 8000 kg | 20533 kg | 28,533 |
| Fossiguano | 4222 | 8000 kg | 20533 kg | 28,533 |

In Meleguano, Pavesi found, after careful examination, that the population consumed each week per head:

| | |
|---|---|
| Rice or corn in soup | 1,332 grams |
| Corn bread | 1,350 grams |
| Beans | 232 grams |
| Fat | 332 grams |
| Vegetables | 133 grams |
| Other starchy materials | 992 grams |
| Proteid substances | 160 grams |

According to the facts noted in the report of Frinli, by the Engineer Cannis: "in a population which numbers three per cent. of Pellagrins, each peasant consumes annually, besides 372 kilograms of corn, from ten to ninety kilograms of each of the following: beans, rice, potatoes, and other vegetables, pork and bacon, olive oil, fish, chickens and wine. They do not eat beef except on festal days, for a marriage or an illness, or when an animal of the herd dies.

All of which goes to show that the peasants of northern Italy can afford as an article of common diet oil; whether olive or not is another question. This is sufficient to show that objection two is not based upon fact.

In Statements two and three the Secretary shows that he has only an incorrect acquaintance with the theory that he is undertaking to show is untenable. He shows clearly that he does not know that olive oil is not included in the list of oils that have been suspected of causing Pellagra. The facts that he mention here are strong points in favor of the theory and one which explains the geographical distribution of Pellagra in this region. Even the poorer classes of central Italy eat mostly olive oil or olive nut oil and because of this are not frequently affected by Pellagra.

The Secretary shows so clearly that he is ignorant in regard to his subject that it seems hardly necessary to discuss his other objections. However, the reader will be given the opportunity of taking his choice.

4. "In Roumania, which has for a long time been horribly scourged by Pellagra, oils are never eaten by the peasants." While this statement may be true, it does not seem to be even reasonable. The price of olive oil may be the basis for this statement, but it would be extraordinary if these people have not long ago followed the example of their neighbors in Austria, Hungary and Southern Russia, and to meet the stringency of circumstances, produced comestible oil from the oleogenous seed which surround them.

5. "In Southern France, where Pellagra was at one time endemic, the food of the peasants has remained the same, with the exception only that what corn is eaten is now harvested and preserved with extreme care, with the result that Pellagra is no longer known in these provinces." The answer in part to this statement will be found in Journal-Record of Medicine, March, 1912.

Marie states that Savoy, after its annexation to France, kept up for a long time the dietetic habits of northern Italy, but the use of corn meal has declined since 1860, and in the poor districts of the Pyrennees and the Garonne basin and in the Landes, where Spanish influence prevails, the use of a corn dietary persisted up to the middle of the nineteenth century.

Discussion as to the dietetic changes incident to French influences and the important changes in the amelioration in the condition of the peasantry and the reports of Mr. Brodie in regard to the consumption of oils, shows that the food of these people has not remained the same. Hence this statement will have to be excluded in forming conclusions.

*Plate 1, Stage of Erythema. Winter Pellagra.*

6. "Oils are more or less used all over northern Europe and the northern portions of North America, and Pellagra is there practically unknown.

8. "Cotton seed oil is mixed with lard and used quite as much in the northern portions of the United States, in Canada, and some countries of northern Europe, as it is in Georgia, with no ill effects."

For the present objection seven is passed, because six and eight are practically the same.

*Plate 2. Same case as Plate 1. Not a pellagrous lesion but due to heat from open fire.*

The reports show that Pellagra is rapidly spreading over the northern portion of the United States and is in direct proportion to the amount of cotton seed oil consumed. No one who is posted, not even representatives of the cotton seed oil industry, will deny that in the cotton belt and in Illinois, where Pellagra prevails, by far more cotton seed oil is consumed than in any region on earth. There are no statistics to show the relative consumption by States, but I am informed, by one who knows, that more cotton seed oil is consumed in Georgia than in any other State in the Union. Illinois being next in the amount consumed, does not show that the per capita consumption is as great as in South Carolina or Alabama.

Some figures given in the Journal-Record of Medicine, Mar., 1912, show that the amount of cotton seed oil consumed in northern Europe is relatively small and that statements to the contrary are not supported by facts. It should be borne in mind that there are other uses for oil than for eating purposes and other reasons for crushing seed than for obtaining oil for eating purposes. In England, oil seed have been crushed for the oil cake for cattle feeding rather than for the oil. These statements will be further discussed with statement seven, which is as follows:

7. "Cotton seed oil has been used more or less as a food in the United States since 1870, and to a very large extent for the last twenty years, and every practicing doctor knows that Pellagra has only been common among us for a very short time."

The figures given below indicate the amount of oil used for both edible and industrial purposes. There are no statistics to show the proportion used for various purposes:

| Year ending June 30th: | Oil Produced: | Oil Exported: | Oil Retained for home consumption: |
|---|---|---|---|
| | Gallons: | Gallons: | Gallons: |
| 1872 | 2,108,000 | 547,165 | 1,560,835 |
| 1880 | 9,416,000 | 6,977,796 | 2,418,204 |
| 1882 | 11,785,000 | 713,549 | 11,071,451 |
| 1892 | 42,737,000 | 13,859,278 | 28,877,722 |
| 1902 | 119,000,000 | 23,042,848 | 85,957,152 |
| 1907 | 175,724,840 | 41,880,304 | 133,844,536 |

The following, taken from the Year Book of the Department of Agriculture for 1903, discusses the extent of oil consumption (for edible purposes) in this country:

"A distinctive characteristic of the American people, though modified in recent years, has been the use of animal fats both in domestic and industrial life, for many purposes for which the inhabitants of other countries largely used vegetable oils. In domestic life there has always been in the minds of the American housewife a somewhat inexplicable prejudice against the use of vegetable oil for cooking purposes, and until recent years lard has completely usurped the functions here that from remote periods has been accorded in many countries to vegetable oils. That this prejudice is being gradually modified there is no doubt, but it is a tribute to its persistency that vegetable cooking oil even now gains surreptitious access to the American kitchens only under the guise of packages and labels suggestive of lard."

According to the United States Government census of 1907, there were in operation the following number of cotton seed oil mills:

| | | | |
|---|---|---|---|
| United States | 786 | Russia | 6 |
| England | 25 | France | 5 |
| Egypt | 7 | Germany | 2 |
| India | 1 | Mexico | 4 |
| China | 10 | S. America | 27 |

This shows the total number of cotton seed oil mills in northern Europe is twenty-seven, or about one to twenty-nine in the United States. As the United States has been exporting less than one-third of her output, which is scattered over the whole world, it is evident that no country of northern Europe or Canada can consume cotton seed oil to the same extent as it is in Georgia.

France, in round numbers, imports about 1,000,000 gallons of cotton seed oil annually, which when used for edible purposes is always sold admixed with olive oil and peanut oil as salad or table oil. When this amount is compared with the total amount of edible fats used in France, it is seen to be relatively small and is only equal to a hundred and fiftieth part of the amount consumed in the United States. The reader is referred to Journal-Record of Medicine, Mar., 1912, for the discussion relative to the proportionate amount of fats consumed by northern and southern people. It is a significant fact that all northern people coming south, and northern traveling men, comment upon the greasy food set before them in this section.

Furthermore, cotton seed oil consumption in northern states is far behind the south and in direct proportion to the prevalence of pellagra in both sections. Note the increased consumption of oil as shown in the table above.

Objection 8 states that cotton seed oil is mixed with lard and used quite as much, etc. In former years, most of the cotton seed oil consumed was made up in this way, but of recent years the lard has been left out and the usual compound lard and fancy cooking fats (hogless lards, etc.), are now composed entirely of cotton seed oil (75 to 85%) and stearin (15 to 25%.) Pure cotton seed oil as such has come into general use only within the past few years, also winter oil, i. e., cotton seed oil from which the stearin has been removed. It may be significant in this connection that the western states that consume the same "poorly harvested and immature corn" which they ship are lard-producing states and at the same time have reported only sporadic cases of Pellagra or none at all. One exception is Illinois, which ranks next to Georgia in oil consumption, and in this western corn-producing state Pellagra is quite common in State Institutions, where corn is selected and freshly ground.

It should be borne in mind that fat of the class of cotton seed oil may be consumed admixed with other fats in such proportions as to be harmless. What this proportion is remains to be determined. Taking these facts into consideration, together with the fact that unmixed cotton seed oil has come into general use during the last few years and that it may take several years to develop the disease, it will be seen that the consumption of oil and development of Pellagra are historically parallel.

401-3 Empire-Life Bldg.

(*To be Continued.*)

## Figures and Tables

**Plate 1 f1:**
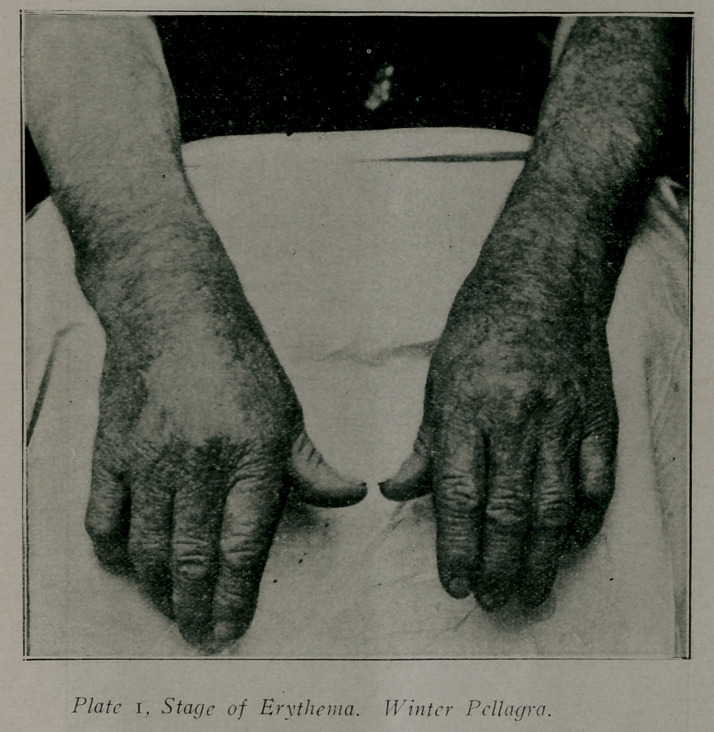


**Plate 2 f2:**